# Explainable AI-Assisted Label-Free Raman Biosensing Reveals Therapy-Associated Spectral Signatures in Melanoma Tumors

**DOI:** 10.3390/bios16090501

**Published:** 2026-09-08

**Authors:** Muhammad Nouman Khan, Qingsong Zhou, Jiaqing Guo, Asif Khalid, Rui Hu

**Affiliations:** Key Laboratory of Optoelectronic Devices and Systems of Guangdong Province and Ministry of Education, College of Physics and Optoelectronic Engineering, Shenzhen University, Shenzhen 518060, China

**Keywords:** label-free Raman spectroscopy, machine learning, explainable artificial intelligence, spectral signature, Melanoma

## Abstract

Sensitive detection of treatment-associated Raman spectral alterations in tumor tissues remains challenging, particularly when such changes are not readily apparent from conventional morphological evaluation. Here, we developed a label-free Raman biosensing strategy combined with explainable machine learning to characterise treatment-associated spectral signatures in melanoma tumours. A B16-F10 melanoma-bearing mouse model was used to compare untreated and PBS-treated controls with cohorts receiving immune checkpoint blockade, anti-angiogenic intervention, or combination therapy. Raman spectra were acquired from multiple spatial regions of melanoma tissues and analyzed using nonlinear dimensionality reduction, supervised classification, and SHAP-based feature interpretation. Although cohort-averaged spectra showed substantial overlap, multivariate analysis revealed treatment-dependent spectral organization, with the combination-treatment cohort showing the most compact and distinguishable spectral profile. Supervised models, including convolutional neural networks, support vector machines, and k-nearest neighbors, further supported the reproducibility of treatment-associated Raman signatures when evaluated using mouse-level validation strategies. SHAP analysis identified discriminative Raman features mainly located within lipid, phospholipid, ester, protein, and collagen-associated vibrational domains, suggesting potential contributions from metabolic- and extracellular-matrix-related biochemical components to treatment-associated spectral discrimination. These findings indicate that Raman spectroscopy integrated with explainable machine learning provides a sensitive, label-free method for distinguishing treatment-associated spectral differences among melanoma tissues. The proposed approach may serve as a complementary spectroscopic tool alongside conventional histological and molecular analyses for investigating treatment-associated tissue-state alterations.

## 1. Introduction

Quantitative characterization of treatment-associated Raman spectral alterations in complex tissues remains a significant analytical challenge. Melanoma is one of the most aggressive skin malignancies, and its global incidence continues to rise, posing an increasing public health burden [1]. Globally, skin cancers accounted for approximately 1.5 million new cases in 2022, including 330,000 melanomas causing nearly 60,000 deaths, with melanoma incidence projected to increase by more than 50% between 2020 and 2040 [2,3]. These trends highlight the urgent need for improved melanoma management and more effective strategies for monitoring therapeutic responses [4,5].

Although immune checkpoint therapies improve melanoma outcomes, reliable characterization and monitoring of treatment-associated biological changes remain challenging [6]. While early-stage melanoma can often be cured by surgical excision, advanced metastatic disease typically requires systemic therapies, including immunotherapy and targeted treatment [7,8]. Accurate early detection and reliable monitoring biomarkers remain limited, as dermoscopy shows modest accuracy, histopathology requires invasive biopsy, and predictive immunotherapy markers are still lacking [9,10,11].

Despite extensive efforts, predictive biomarkers such as PD-L1 expression remain clinically inconsistent due to assay variability and weak correlation with therapeutic outcomes [12]. Alternative biomarkers, including tumor-infiltrating lymphocytes, liquid biopsies, and imaging approaches, remain insufficient for routine clinical application [13,14,15,16,17], highlighting the need for tools capable of resolving therapy-associated biological remodeling.

The tumor microenvironment (TME) plays a critical role in melanoma progression and therapeutic response [18]. Alterations in collagen organization and lipid metabolism are closely associated with immune evasion and therapy resistance [19,20,21,22,23]. However, current technologies remain inadequate for resolving dynamic biochemical remodeling within the TME during treatment. Raman spectroscopy offers a label-free optical approach capable of probing molecular composition through vibrational signatures and has been widely applied in cancer and biomedical analysis [24,25,26,27]. Compared with conventional histology and imaging, Raman spectroscopy provides rapid, label-free biochemical characterization with molecular specificity. Combined with machine learning, it can uncover subtle treatment-associated molecular variations not readily detectable by conventional analysis.

In this study, label-free Raman spectroscopy combined with nonlinear manifold learning and supervised machine learning was used to investigate treatment-associated Raman spectral alterations in melanoma. The study further evaluated whether Raman spectral profiles could distinguish six therapeutic and control conditions and identified model-important Raman shifts using SHAP analysis. The biochemical assignments of these spectral regions were interpreted cautiously on the basis of established Raman literature.

## 2. Materials and Methods

### 2.1. Melanoma Cell Culture and Mice Implantation

**Figure 1:**
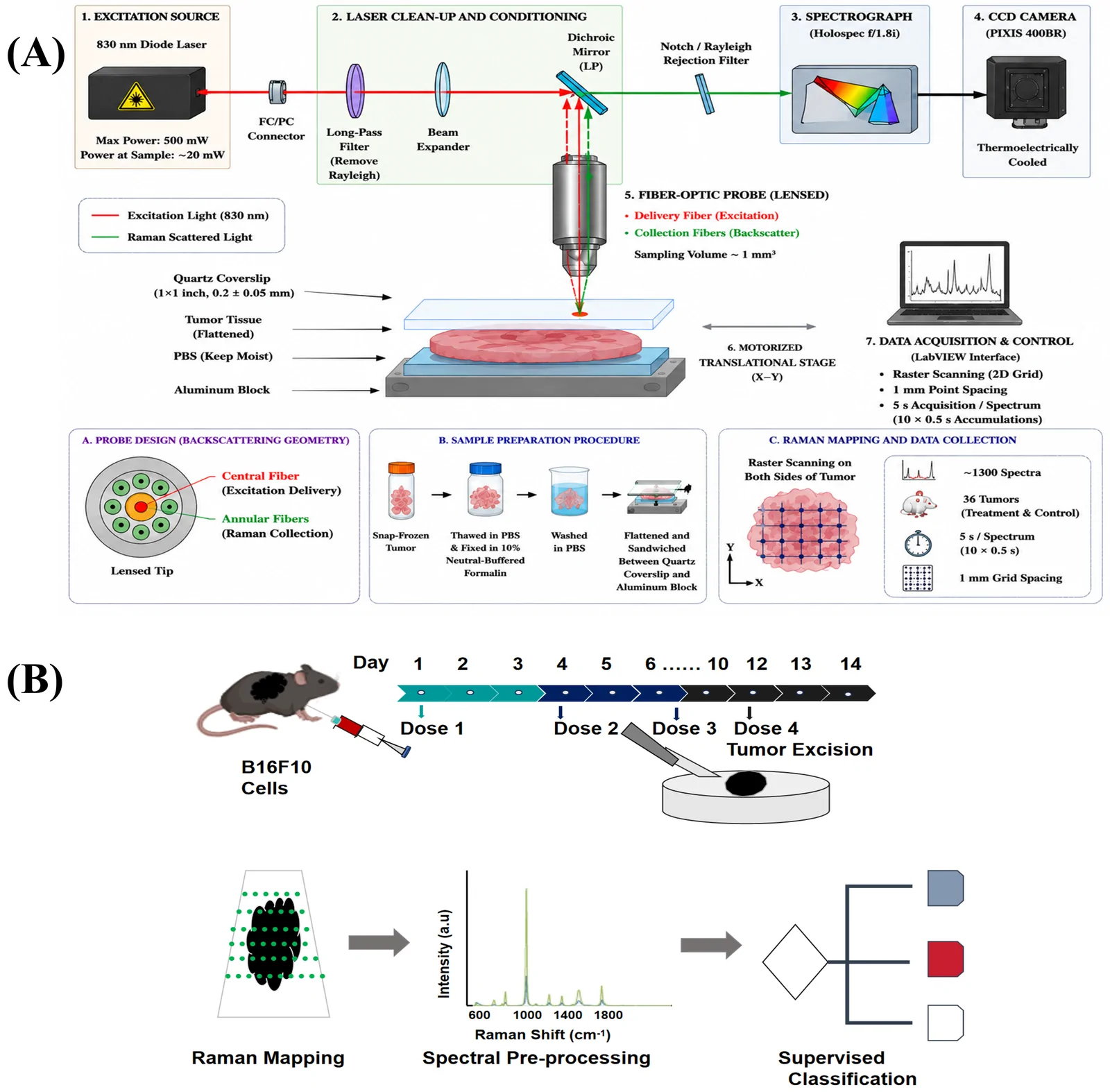
Experimental Raman spectroscopy workflow and representative spectral analysis of melanoma tissues. (**A**) Schematic diagram of the custom-built fiber-probe Raman spectroscopy system and tissue preparation procedure used for label-free Raman spectral acquisition from excised melanoma tissues. (**B**) Treatment and analysis workflow.

Female C57BL/6 mice (4–5 weeks old) were obtained from the Guangdong Medical Laboratory Animal Center under approved ethical protocols (C202110-01). B16-F10 melanoma cells were subcutaneously implanted into the right flank of each mouse. Tumor growth and body weight were monitored throughout the study. Once tumors reached approximately 10–15 mm^2^, mice were randomized into six treatment groups receiving immunotherapy, angiogenesis inhibitors, or combination therapy. One week after the final treatment, tumors were excised following euthanasia and processed for histological evaluation and Raman spectroscopic analysis as shown in Figure 1A,B.

### 2.2. Treatment Design

When tumors reached approximately 10–15 mm^2^, mice were randomized into six cohorts (*n* = 6 tumors per cohort): S1, immune checkpoint inhibitor treatment consisting of anti-CTLA4, anti-PD-1, and anti-PD-L1 antibodies (100 μg each); S2, anti-angiogenic intervention consisting of bevacizumab and anlotinib (100 μg each); S3, PD-1/PD-L1 blockade consisting of anti-PD-1 and anti-PD-L1 antibodies (100 μg each); S4, PBS-treated control; S5, untreated control; and S6, combination treatment consisting of the immune checkpoint inhibitor regimen used in S1 together with the anti-angiogenic intervention used in S2. S1 represented broader multi-checkpoint blockade involving the CTLA-4 and PD-1/PD-L1 axes, whereas S3 was restricted to PD-1/PD-L1 blockade without anti-CTLA-4 treatment. The PBS-treated group (S4) served as a vehicle control to account for potential effects associated with intraperitoneal injection, handling, and vehicle administration, whereas the untreated group (S5) represented the natural progression of the tumor without intervention. Including both control groups enabled assessment of any potential injection-related contributions to the Raman spectral profiles. All treatments were administered by intraperitoneal injection every four days for a total of five doses. One week after the final treatment, mice were euthanized, and tumors were harvested for histological evaluation and Raman spectroscopic analysis.

### 2.3. Tissue Slice Preparation

Excised melanoma tumors were fixed, embedded, sectioned, and mounted on quartz substrates for Raman spectroscopic measurements. Before spectral acquisition, FFPE tissue sections were deparaffinized using standard procedures and air-dried under controlled conditions. The use of quartz substrates minimized background interference during Raman acquisition. Multiple spatially distinct tumor regions were selected from each tissue section to account for intratumoral heterogeneity and to reduce sampling bias.

### 2.4. Raman Spectroscopy

Raman measurements were performed on deparaffinized FFPE melanoma tissue sections mounted on quartz substrates. Spectra were acquired using a custom-built fiber-probe Raman system equipped with an 830 nm diode laser, spectrograph, and thermoelectrically cooled CCD detector. Raman mapping was conducted through raster scanning with 1 mm spacing across spatially distinct tumor regions. A total of approximately 1300 Raman spectra were initially acquired from 36 melanoma tumors. Following spectral quality assessment and preprocessing, 1080 high-quality individual Raman spectra were retained, comprising 180 spectra from each of the six experimental groups. These individual spectra were used for the development, validation, and evaluation of the CNN, SVM, kNN, and XGBoost classification models. For exploratory visualization and qualitative comparison, a balanced subset of 120 quality-controlled spectra was selected from each experimental group. Every ten spectra acquired from the same tumor specimen were averaged to generate one representative spectrum, yielding 12 representative spectra per group and 72 representative spectra in total. These averaged spectra were used only for the dimensionality-reduction and PLS-DA analyses presented in in the subsequent figures and were not used for the training, validation, or testing of the predictive classification models. Spectral averaging was performed exclusively among spectra acquired from the same tumor specimen, and no representative spectrum combined measurements from different animals.

### 2.5. Machine Learning and Multivariate Spectral Analysis

Raman spectra acquired between 200 and 3200 cm^−1^ were analyzed using complementary unsupervised, supervised, and explainable machine-learning approaches implemented in Python 3.11. Before model construction, spectra were preprocessed by baseline correction, smoothing, intensity normalization, and removal of low-quality spectra according to predefined signal-quality criteria. For Raman spectra preprocessing, asymmetric least-squares (ALS) baseline correction (λ = 10^6^, *p* = 0.001), followed by Savitzky–Golay smoothing (11-point window, second-order polynomial) and vector normalization, was applied. Spectra exhibiting detector saturation, cosmic-ray artifacts, excessive fluorescence, or poor signal-to-noise ratio were excluded prior to machine-learning analysis.

Nonlinear dimensionality-reduction methods, including t-SNE, UMAP, and PHATE, were used to visualize treatment-associated spectral organization, while PLS-DA was used as a supervised multivariate method to assess cohort separation. For dimensionality reduction, t-SNE was implemented with two components, a perplexity of 30, an automatically determined learning rate, PCA initialization, and a random seed of 42. UMAP was performed with 12 nearest neighbors, a minimum distance of 0.08, cosine distance, two components, and a random seed of 42. PHATE was applied with 10 nearest neighbors, a decay parameter of 40, two components, and a random seed of 42, while PLS-DA was conducted using two latent variables. Similarly, supervised classification was performed using one-dimensional convolutional neural networks, support vector machines with a radial basis function kernel, and k-nearest neighbors. Hyperparameters were optimized by grid search within the training data.

To minimize spectrum-level data leakage caused by multiple spectra acquired from the same tumor, model training and evaluation were performed using mouse-level partitioning. Spectra from the same mouse were kept exclusively within either the training or testing set in each validation round. In addition to five-fold cross-validation, leave-one-mouse-out validation was used to assess generalizability across biological replicates. Feature scaling, hyperparameter selection, and model fitting were performed only within the training data. Model performance was evaluated using accuracy, precision, recall, F1-score, confusion matrices, and one-vs-rest receiver operating characteristic analysis. For model interpretation, an XGBoost classifier trained under the same validation framework was analyzed using SHAP values to identify Raman shifts contributing most strongly to treatment classification.

### 2.6. Experimental Workflow for Raman-Based Assessment of Therapy-Associated Spectral Alterations

To investigate therapy-associated Raman spectral alterations in melanoma tissues, a B16-F10 melanoma-bearing mouse model was established and subjected to different therapeutic interventions as described in Section 2.2. After completion of the treatment regimen, tumor tissues were harvested, processed into FFPE sections, and analyzed using label-free Raman spectroscopy. The overall experimental workflow integrated tumor implantation, treatment intervention, tissue preparation, Raman spectral acquisition, multivariate analysis, supervised classification, and SHAP-based model interpretation (Figure 1A,B). This design enabled Raman-based comparison of spectral differences among untreated, PBS-treated, monotherapy-treated, and combination-treated melanoma tissues under a controlled experimental framework. Rather than relying on a single spectral marker, the study aimed to determine whether treatment-associated tissue-state changes could be captured from high-dimensional Raman fingerprints and further interpreted using explainable machine-learning analysis.

## 3. Results

### 3.1. Raman Spectroscopy Reveals Conserved Biochemical Architecture Across Treatment Cohorts

**Figure 2:**
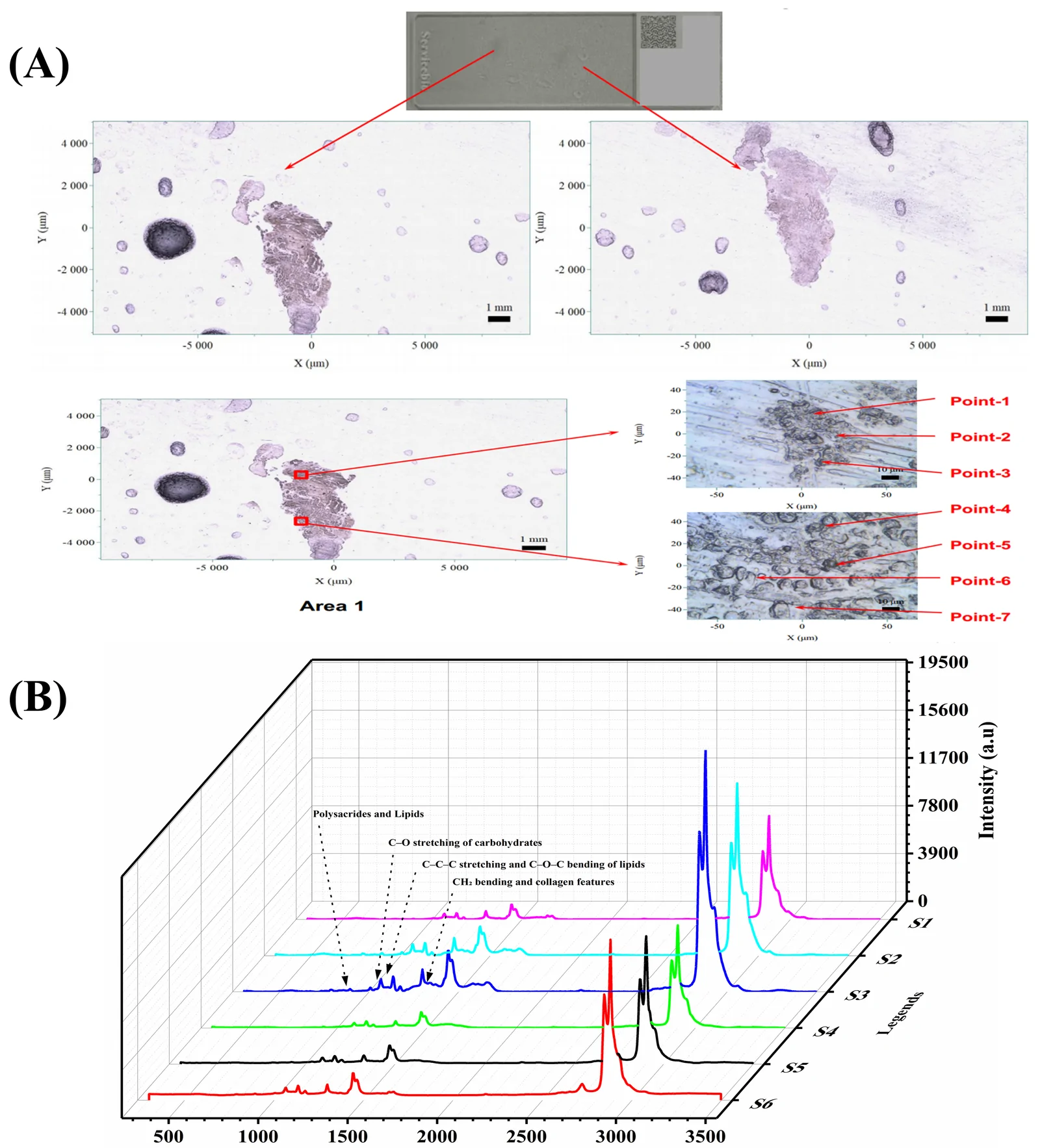
(**A**) FFPE melanoma section showing Raman sampling regions. (**B**) Mean Raman spectra of six treatment cohorts, revealing conserved lipid, protein, carbohydrate, and collagen-associated bands with substantial spectral overlap among groups.

Raman spectra were acquired from multiple spatially distinct regions of each melanoma tissue section to reduce local sampling bias and account for intratumoral heterogeneity (Figure 2A). Approximately 1300 Raman spectra were obtained from 36 tumors across the six cohorts, covering the spectral range of 200–3200 cm^−1^. This sampling strategy enabled analysis of Raman spectral variations from multiple tumor regions while reducing potential local sampling bias.

Mean Raman spectra for all six cohorts are presented in Figure 2B. Across groups, prominent vibrational bands corresponding to major biochemical constituents were consistently observed, including 922.1 cm^−1^ (C–C–C stretching in polysaccharides/lipids), 1034.7 cm^−1^ (C–O stretching of carbohydrates), 1061.6 and 1094.4 cm^−1^ (lipid/protein-associated modes), 1200.9 cm^−1^ (amide III; protein secondary structure), and 1325.8 cm^−1^ (CH_2_ bending; collagen-associated features). The presence of these common spectral features indicates that melanoma tissues from different treatment cohorts retained a broadly conserved biochemical architecture. Similarly, the inclusion of both PBS-treated and untreated control groups allowed evaluation of potential effects associated with the injection procedure itself. The overall similarity between the PBS-treated and untreated control groups suggests that vehicle administration and handling had limited influence on the observed Raman spectral differences.

Direct comparison of cohort-averaged spectra did not reveal clear visual separation among the six groups, suggesting that treatment-associated biochemical differences were subtle and embedded within partially overlapping Raman features. This spectral overlap highlights the limitation of simple mean-spectrum inspection and supports the need for multivariate and machine-learning analyses to extract structured treatment-dependent variance from the Raman dataset. As summarized in Table 1, following spectral quality assessment and preprocessing, the final machine-learning dataset comprised approximately 1080 quality-controlled individual Raman spectra, including approximately 180 spectra from each of the six experimental groups. The equal class sizes ensured that the machine-learning models were trained and evaluated using a balanced dataset. For exploratory visualization and PLS-DA analysis, a balanced subset of 120 quality-controlled spectra was selected from each group. Every ten spectra acquired from the same tumor specimen were averaged to generate one representative spectrum, yielding 12 representative spectra per group and 72 representative spectra in total. These averaged spectra were not used for training or evaluating the predictive classification models.

### 3.2. Multimodal Dimensionality Reduction and Supervised Classification of Raman Spectra

**Figure 3:**
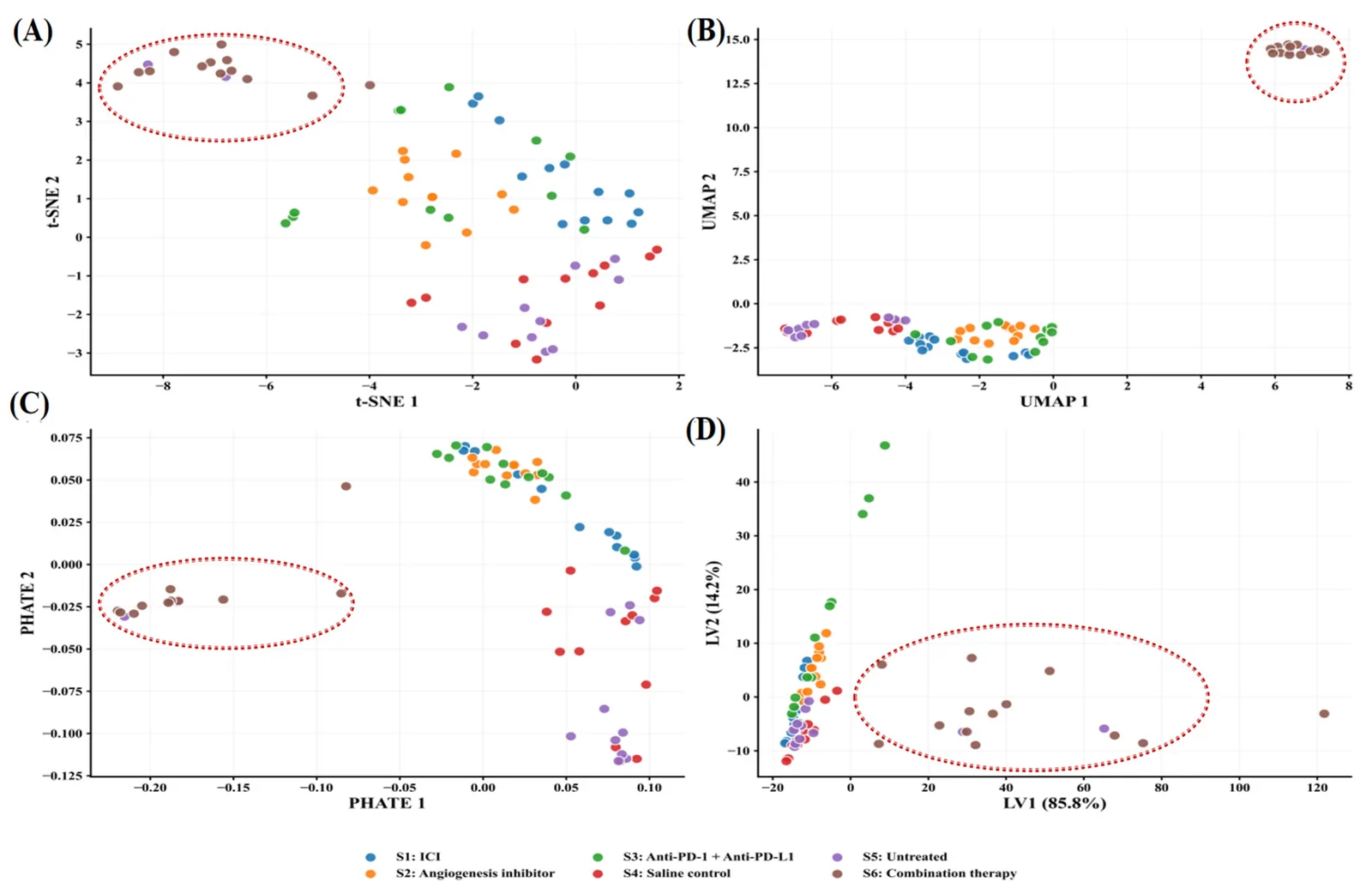
Dimensionality reduction in Raman spectra from the six treatment groups using (**A**) t-SNE, (**B**) UMAP, (**C**) PHATE, and (**D**) PLS-DA. Each point represents one representative spectrum used for analysis (n = 72). t-SNE was performed using a perplexity of 30, an automatic learning rate, PCA initialization, and random seed 42. UMAP was performed with 12 nearest neighbors, a minimum distance of 0.08, cosine distance, and random seed 42. PHATE was implemented with 10 nearest neighbors, a decay parameter of 40, and random seed 42. PLS-DA was performed using two latent variables. The dimensionality reduction methods were used for qualitative visualization only. Model performance was evaluated using grid-search optimization, five-fold cross-validation, and leave-one-mouse-out validation.

To investigate treatment-associated differences in Raman spectral patterns, t-SNE, UMAP, PHATE, and PLS-DA were applied to Raman spectra from six melanoma treatment cohorts (Figure 3). t-SNE and UMAP revealed treatment-dependent spectral organization, with the combination therapy cohort (S6) forming the most compact and distinct cluster, whereas monotherapy and control groups showed partial overlap (Figure 3A,B). PHATE demonstrated continuous transitions in the Raman spectral feature space (Figure 3C). Supervised PLS-DA further confirmed robust cohort discrimination (Figure 3D). Overall, the t-SNE, UMAP, PHATE, and PLS-DA plots showed treatment-associated patterns in the Raman spectral feature space, although partial overlap among groups remained. These visualizations were interpreted qualitatively and were not considered independent evidence of statistically distinct clusters or specific biological mechanisms. The observed manifold organization supported KNN, SVM, and CNN classification performance, indicating that the spectral separation reflected distinguishable Raman spectral patterns.

### 3.3. SHAP-Based Raman Feature Interpretation

**Figure 4:**
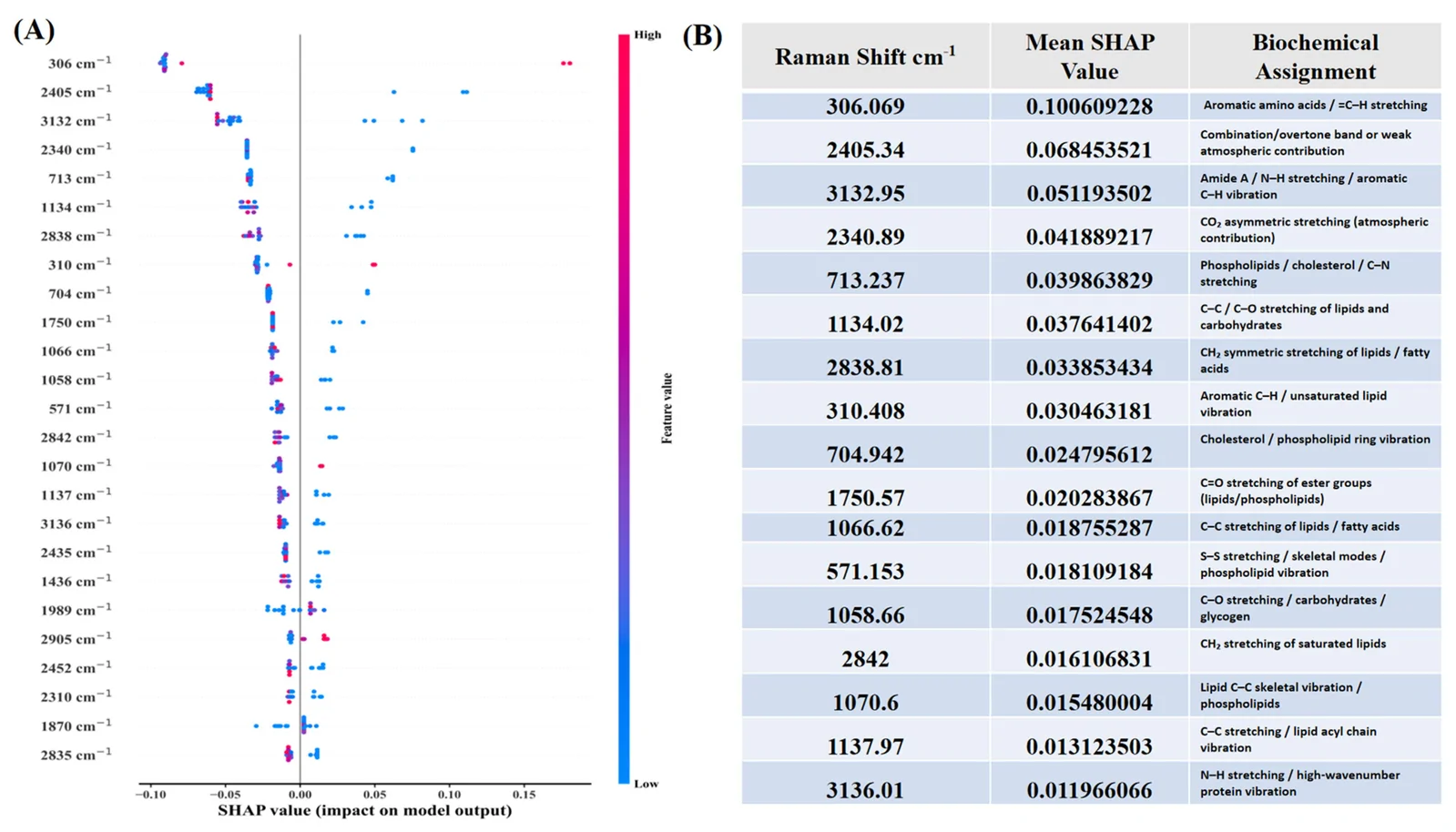
SHAP-based interpretation of Raman spectral features associated with melanoma therapy response. (**A**) SHAP beeswarm plot showing discriminative Raman features. (**B**) SHAP importance profile highlighting dominant lipid, phospholipid, ester, and protein-associated Raman bands contributing to treatment classification. Each point represents the contribution of an individual Raman feature to the model output for one spectrum, while the colour scale indicates the corresponding normalized Raman intensity.

To improve model interpretability and identify treatment-associated Raman spectral features, SHapley Additive exPlanations (SHAP) analysis was performed on the optimized XGBoost classifier as shown in Figure 4A,B. SHAP analysis identified several highly discriminative Raman bands associated with treatment groups. The most influential features included bands near 306, 713, ~1066–1138, ~1750, ~2840, and ~3130 cm^−1^. According to established Raman spectroscopy literature, these bands have been assigned to vibrational modes associated with lipid, protein, and carbohydrate-related spectral regions. These assignments are presented as literature-based interpretations rather than direct experimental confirmation.

The combination therapy cohort exhibited more consistent SHAP feature distributions than the monotherapy and control groups, suggesting that the classification model relied on a more coherent set of discriminative Raman spectral features. Overall, the SHAP analysis identified spectral regions that contributed substantially to machine-learning predictions. The biological interpretation of these spectral features is based on previously reported Raman band assignments and should not be considered direct evidence of therapy-induced molecular or biochemical changes without independent validation.

### 3.4. Convolutional Neural Network Validates Treatment-Specific Spectral Signatures

**Figure 5:**
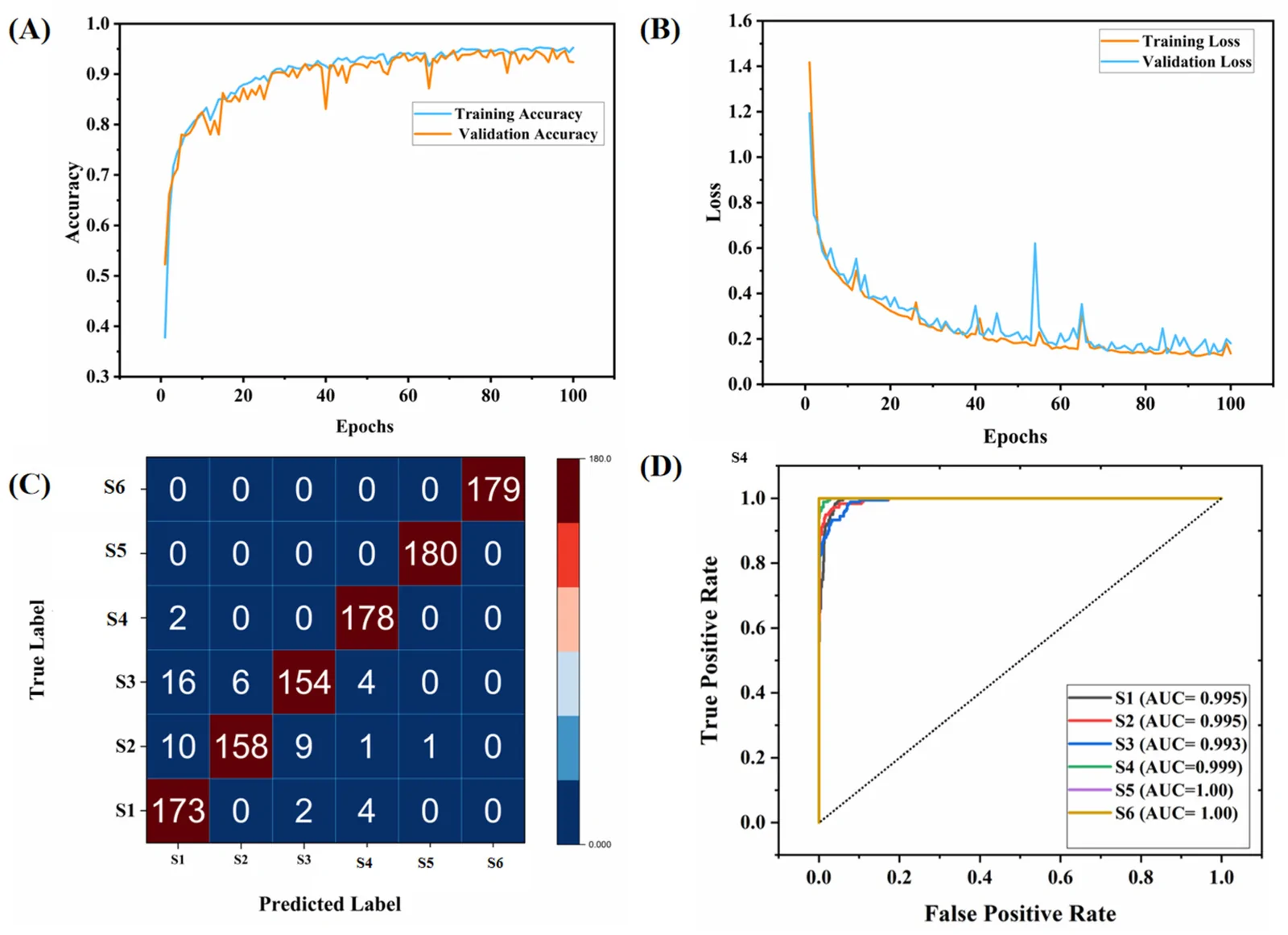
Convolutional neural network (CNN) classification of Raman spectra. (**A**) Training and validation accuracy curves, (**B**) training and validation loss curves, (**C**) confusion matrix, and (**D**) receiver operating characteristic (ROC) curves for all six cohorts. The CNN achieved rapid convergence (~30 epochs) and strong classification performance with minimal misclassifications.

To evaluate treatment-specific spectral discrimination, a convolutional neural network (CNN) was applied to classify Raman spectra from the six cohorts (Figure 5). The model rapidly converged, achieving stable training and validation performance within ~30 epochs (Figure 5A), while loss curves stabilized without evident overfitting (Figure 5B). Confusion matrix analysis demonstrated high classification accuracy with minimal misclassification among groups (Figure 5C). ROC curves further confirmed strong discriminative performance across all cohorts, with the combination therapy group (S6) showing the greatest separability (Figure 5D). These findings indicate that CNN classification captured reproducible spectral patterns associated with different experimental groups.

### 3.5. Conventional Supervised Models Corroborate Therapy-Driven Spectral Discrimination

**Figure 6:**
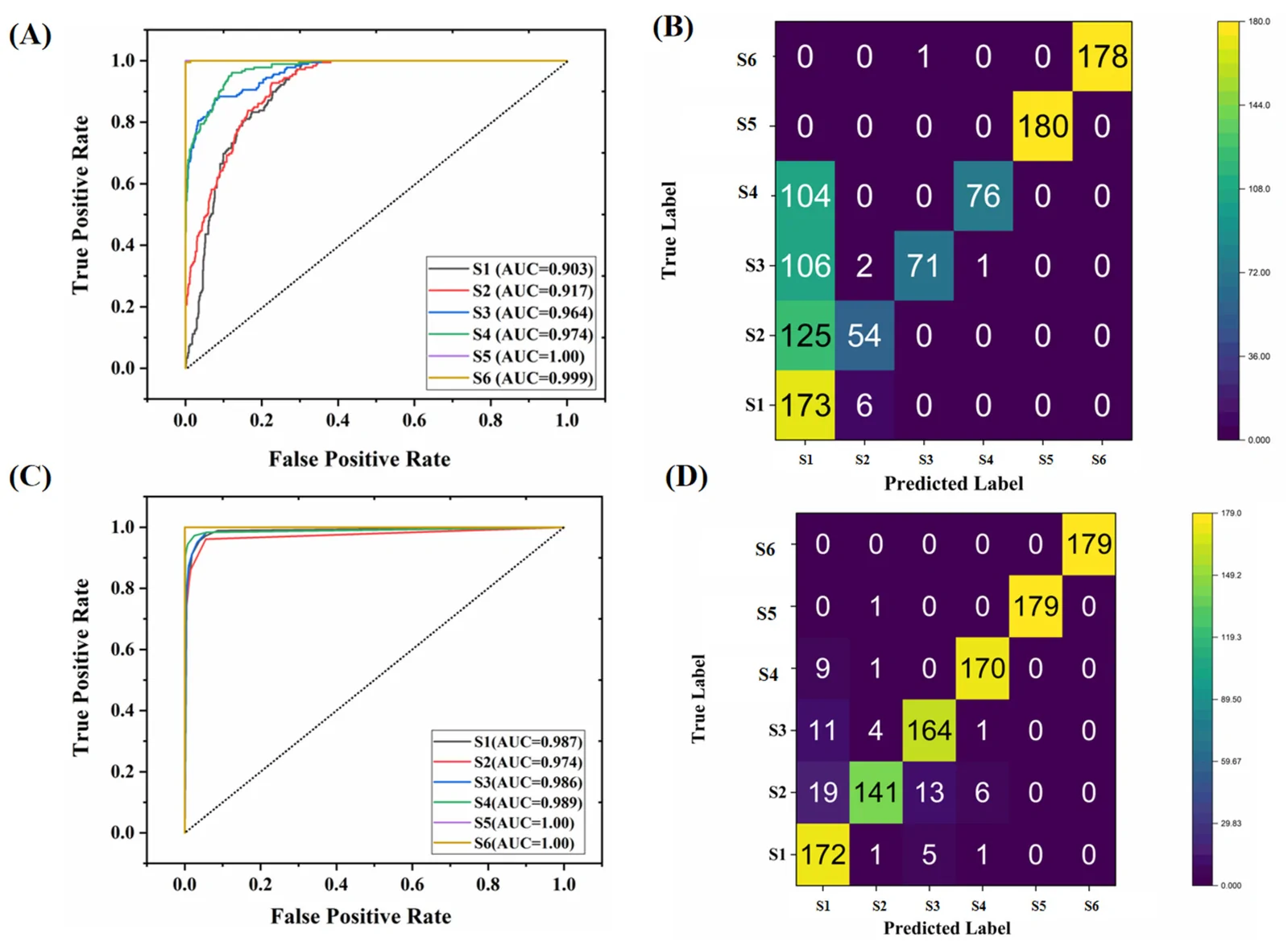
Supervised machine learning classification using support vector machine (SVM) and k-nearest neighbors (kNN). (**A**) ROC curves of SVM classification, (**B**) SVM confusion matrix, (**C**) ROC curves of kNN classification, and (**D**) kNN confusion matrix. Both models achieved robust classification across cohorts, with S6 consistently distinguished as the most separable group.

To further evaluate treatment-specific spectral separation, SVM and kNN classifiers were implemented using the same preprocessing and validation strategy as the CNN model (Figure 6). SVM demonstrated strong classification performance with high cohort precision, particularly for the combination therapy group (S6), which showed clear separability (Figure 6A,B). Similarly, kNN achieved robust discrimination, although minor overlap persisted among certain monotherapy and control cohorts (Figure 6C,D). These classification patterns were consistent with the spectral organization observed in t-SNE, UMAP, PHATE, and PLS-DA analyses. Collectively, the results demonstrate that therapy-associated Raman signatures are reproducible, structurally encoded, and robust across multiple machine-learning frameworks.

### 3.6. Integrated Analysis of Treatment-Associated Raman Spectral Signatures

Integrating unsupervised and supervised analyses, reproducible treatment-specific Raman signatures were identified across all six cohorts. The combination therapy group (S6) consistently exhibited the most compact and separable spectral organization across t-SNE, UMAP, PHATE, PLS-DA, SHAP, CNN, SVM, and kNN analyses, indicating coordinated treatment-associated spectral changes related to biochemical components within the tumor microenvironment. The dominant discriminative Raman bands were associated with lipid- and collagen-related regions, suggesting therapy-dependent modulation of metabolic and extracellular matrix organization. These findings suggest that Raman spectroscopy may provide a sensitive label-free approach for detecting subtle molecular-level tissue-state changes that are not readily apparent from cohort-averaged spectra.

## 4. Discussion

Raman spectroscopy provides label-free biochemical fingerprints, but it does not directly identify specific metabolites, proteins, or extracellular matrix components. The treatment-associated spectral features observed in this study are consistent with previously reported biochemical alterations in the tumor microenvironment following therapeutic intervention; however, direct correspondence between individual Raman bands and specific biomarkers cannot be established from the present data alone. Future studies integrating Raman spectroscopy with targeted metabolomics, immunohistochemistry, collagen imaging, or spatial molecular profiling will enable more comprehensive biological validation and help establish direct relationships between Raman spectral features, molecular biomarkers, and pathological endpoints. In this study, we demonstrated that label-free Raman spectroscopy, combined with nonlinear dimensionality reduction, supervised machine learning, and SHAP-based feature interpretation, can sensitively capture treatment-associated spectral signatures in melanoma tumors. Although the cohort-averaged Raman spectra showed substantial overlap among treatment groups, multivariate analysis revealed structured spectral organization, with the combination-treatment cohort exhibiting the most compact and distinguishable profile. These findings suggest that Raman spectroscopy provides a sensitive optical biosensing strategy for detecting subtle tissue-state changes that may not be readily resolved by direct spectral inspection or conventional gross evaluation. A key observation of this work is that treatment-associated spectral discrimination was consistently supported by multiple analytical strategies. Nonlinear manifold learning methods, including t-SNE, UMAP, and PHATE, revealed therapy-dependent organization of Raman spectral features, whereas supervised models further confirmed that treatment-specific signatures were reproducible at the individual-spectrum level. The consistent separability of the combination-treatment cohort across different models suggests that the observed spectral differences were not restricted to a single algorithmic framework. Importantly, model interpretation using SHAP analysis provided additional chemical insight by identifying Raman shifts associated mainly with lipid-, phospholipid-, ester-, protein-, and collagen-related vibrational domains. Thus, the value of the present approach lies not only in classification performance, but also in its ability to connect machine-learning predictions with chemically interpretable Raman features. In Raman-based classification studies, discriminative peaks and intensity ratios are more appropriately regarded as spectral markers or molecular signatures, because their designation as biological biomarkers requires independent analytical and biological validation [28].

The lipid-associated Raman features identified in this study may reflect treatment-related changes in the metabolic state of the tumor microenvironment. Lipid metabolism is increasingly recognized as an important determinant of tumor progression, immune regulation, and response to immune checkpoint blockade [21,29]. Within the tumor microenvironment, tumor cells, immune cells, and stromal components compete for nutrients and undergo extensive metabolic adaptation. Changes in fatty-acid utilization, phospholipid composition, and membrane-associated biochemical states can influence immune-cell function, tumor-cell survival, and therapeutic response. In this context, the discriminative Raman bands assigned to lipid- and ester-related domains may indicate that combination treatment induced a more coordinated metabolic remodeling pattern than monotherapy or control conditions. However, because Raman bands represent composite biochemical signatures rather than single-molecule readouts, these assignments should be interpreted as chemically informed spectral annotations rather than direct molecular validation of specific lipid species.

In addition to lipid-related features, collagen-associated Raman components also contributed to treatment discrimination. The extracellular matrix is now understood as an active regulator of tumor immunity, rather than a passive structural scaffold. Dense collagen deposition and fibrosis can physically impede T-cell trafficking and create hypoxic niches that promote PD-L1 expression and immunosuppressive signaling [30,31]. Preclinical studies have demonstrated that targeting stromal pathways can sensitize tumors to checkpoint therapy. For example, inhibition of focal adhesion kinase reduced fibrosis and enhanced T-cell infiltration in pancreatic cancer models [32]. Similarly, blockade of CXCR4 alleviated desmoplasia and improved immunotherapy efficacy in metastatic breast cancer [33]. Therefore, the contribution of collagen-related Raman features to cohort separation may reflect treatment-associated changes involving extracellular matrix-related components. In particular, the compact clustering of the combination-treatment cohort may reflect a more coherent biochemical state involving both metabolic and extracellular-matrix-associated changes. Nevertheless, Raman spectroscopy alone cannot directly resolve collagen architecture, fiber orientation, or matrix mechanics. The collagen-related spectral variations observed in this study should therefore be interpreted as treatment-associated molecular signatures rather than direct evidence of tissue remodeling. Recent studies have demonstrated that Raman spectroscopy is a powerful tool for identifying treatment-associated molecular fingerprints; however, careful biological interpretation and independent validation remain essential when relating spectral variations to the underlying biochemical processes [34]. Therefore, complementary histological, biochemical, or imaging analyses will be necessary to validate the biological significance of the observed spectral changes.

The integration of explainable machine learning is particularly important for Raman-based biosensing applications. High-dimensional Raman spectra contain overlapping biochemical information, and classification models may achieve strong performance without revealing the molecular basis of discrimination. By combining supervised classification with SHAP analysis, this study identified spectral regions that contributed most strongly to treatment classification and provided a more transparent interpretation of model outputs. Such explainability is essential for developing Raman spectroscopy from a pattern-recognition tool into a more reliable analytical platform for biological and translational applications. In this sense, the present workflow offers a framework for Raman-based tissue-state characterization in which spectral classification and biochemical interpretability are considered together. Several limitations should be acknowledged. First, this study was performed in a mouse melanoma model, and validation in independent cohorts and human melanoma specimens will be necessary before clinical translation can be considered. Second, although multiple Raman spectra were collected from spatially distinct regions of each tumor to better capture intratumoral variability, the acquired spectra were not registered with corresponding histopathological sections. Consequently, some spectral variation may reflect differences in local tissue composition, such as viable tumor, stromal components, necrotic regions, or inflammatory infiltrates, in addition to treatment-related molecular changes. Therefore, the potential influence of tissue heterogeneity on the classification results cannot be completely excluded. Furthermore, because multiple spectra acquired from the same biological specimen are inherently correlated, strict mouse-level validation and independent biological replication remain essential for assessing the robustness and generalizability of machine-learning models. Future studies integrating Raman spectroscopy with histopathological co-registration or multimodal imaging will enable more precise interpretation of treatment-associated spectral signatures. Third, Raman spectroscopy provides label-free biochemical fingerprints, but it does not directly identify individual metabolites, proteins, or extracellular matrix components. Future studies combining Raman spectroscopy with targeted metabolomics, immunohistochemistry, collagen imaging, or spatial molecular profiling would further clarify the biological basis of the discriminative spectral features. Importantly, the present study was based exclusively on endpoint tissue samples collected after treatment; therefore, our findings demonstrate treatment-associated molecular signatures rather than the capability for early therapeutic assessment. Longitudinal in vivo or minimally invasive Raman measurements will be necessary to determine whether this approach can detect treatment response at earlier stages and monitor dynamic therapeutic changes over time.

## 5. Conclusions

In summary, this study demonstrates that label-free Raman spectroscopy combined with machine-learning analysis can distinguish treatment-associated spectral patterns among melanoma tissues subjected to six therapeutic and control conditions. Animal-grouped data partitioning was used to reduce spectrum-level leakage, and SHAP analysis identified Raman shifts that contributed strongly to the XGBoost model outputs. However, the biochemical assignments of these spectral features remain tentative and literature-based, and independent histopathological and molecular validation will be required to establish their biological origins. The proposed strategy should therefore be considered a complementary spectroscopic approach rather than a standalone mechanistic or clinically validated assay.

## Figures and Tables

**Table 1:** Distribution of spectra used for machine-learning analysis.

Group	Mice (n)	Acquired Spectra	Quality-Controlled Spectra Used for ML	Spectra Per Averaged Sample	Representative Averaged Spectra (Visualization Only)
S1	6	214	180	10	12
S2	6	218	180	10	12
S3	6	220	180	10	12
S4	6	216	180	10	12
S5	6	216	180	10	12
S6	6	216	180	10	12
Total	36	1300	1080	—	72

## Data Availability

Data will be made available upon request.
